# Health Tract, No. 23

**Published:** 1865

**Authors:** 


HEALTH TRACT, No. 23.

### BURNS AND BITES.

### POISONS.

For any poison, the most speedy, certain, and most frequently efficacious remedy in the world, if immediately taken, is a heaping teaspoonful of ground mustard, stirred rapidly in a glass of cold water, and drank down at a draught, causing instantaneous vomiting. As soon as the vomiting ceases, swallow two tablespoonfuls or more of sweet-oil, or any other mild oil.

If no ground mustard is at hand, drink a teacupful or more of sweet-oil, or any other pure mild oil, melted hog's lard, melted butter, train oil, cod-liver oil, any of which protect the coats of the stomach from the disorganizing effects of the poison; and, to a certain extent, by filling up the pores of the stomach, (the mouths of the absorbents,) prevent the poison being taken up into the circulation of the blood. Persons bitten by rattlesnakes have drank oil freely, and recovered. These are things to be done while a physician is being sent for.

### BITES AND STINGS.

Apply instantly, with a soft rag, most freely, spirits of hartshorn. The venom of stings being an acid, the alkali nullifies them. Fresh wood ashes, moistened with water, and made into a poultice, frequently renewed, is an excellent substitute—or, soda or saleratus—all being alkalies.

To be on the safe side, in case of snake or mad-dog bites, drink brandy, whisky, rum, or other spirits, as free as water—a teacupful, or a pint or more, according to the aggravation of the circumstances.

### POULTICES.

As to inflammation, sores, cuts, wounds by rusty nails, etc., the great remedy is warmth and moisture, because these promote evaporation and cooling; whatever kind of poultice is applied, that is best which keeps moist the longest, and is in its nature mild; hence cold light (wheaten) bread, soaked in sweet milk, is one of the very best known. There is no specific virtue in the repulsive remedy of the " entrails of a live chicken," of scraped potatoes, turnips, beets, carrots, or any other scrapings; the virtue consists in the mild moisture of the application. Hence the memory need not be burdened with the recollection of particular kinds of poultices, but only with the principle that that poultice is best which keeps moist longest without disturbance.

### SCALDS AND BURNS.

The best, most instantaneous, and most accessible remedy in the world, is to thrust the injured part in cold water, send for a physician, and while he is coming, cover the part an inch or more deep with common flour. The water gives instantaneous relief by excluding the oxygen of the air; the flour does the same thing, but is preferable, because it can be kept more continuously applied, with less inconvenience, than by keeping the parts under water. As they get well, the flour scales off, or is easily moistened and removed. If the injury is at all severe, the patient should live mainly on tea and toast, or gruels, and keep the bowels acting freely every day, by eating raw apples, stewed fruits, and the like. No better and more certain cure for scalds and burns has ever oeen proposed.

